# Perivascular Adipose Tissue as a Relevant Fat Depot for Cardiovascular Risk in Obesity

**DOI:** 10.3389/fphys.2018.00253

**Published:** 2018-03-21

**Authors:** Rafael M. Costa, Karla B. Neves, Rita C. Tostes, Núbia S. Lobato

**Affiliations:** ^1^Department of Pharmacology, Ribeirao Preto Medical School, University of São Paulo, Ribeirao Preto, Brazil; ^2^Institute of Cardiovascular and Medical Sciences, British Heart Foundation, Glasgow Cardiovascular Research Centre, University of Glasgow, Glasgow, United Kingdom; ^3^Institute of Health Sciences, Federal University of Goias, Jatai, Brazil

**Keywords:** perivascular adipose tissue, obesity, vascular function, cardiovascular risk, adipokine

## Abstract

Obesity is associated with increased risk of premature death, morbidity, and mortality from several cardiovascular diseases (CVDs), including stroke, coronary heart disease (CHD), myocardial infarction, and congestive heart failure. However, this is not a straightforward relationship. Although several studies have substantiated that obesity confers an independent and additive risk of all-cause and cardiovascular death, there is significant variability in these associations, with some lean individuals developing diseases and others remaining healthy despite severe obesity, the so-called metabolically healthy obese. Part of this variability has been attributed to the heterogeneity in both the distribution of body fat and the intrinsic properties of adipose tissue depots, including developmental origin, adipogenic and proliferative capacity, glucose and lipid metabolism, hormonal control, thermogenic ability, and vascularization. In obesity, these depot-specific differences translate into specific fat distribution patterns, which are closely associated with differential cardiometabolic risks. The adventitial fat layer, also known as perivascular adipose tissue (PVAT), is of major importance. Similar to the visceral adipose tissue, PVAT has a pathophysiological role in CVDs. PVAT influences vascular homeostasis by releasing numerous vasoactive factors, cytokines, and adipokines, which can readily target the underlying smooth muscle cell layers, regulating the vascular tone, distribution of blood flow, as well as angiogenesis, inflammatory processes, and redox status. In this review, we summarize the current knowledge and discuss the role of PVAT within the scope of adipose tissue as a major contributing factor to obesity-associated cardiovascular risk. Relevant clinical studies documenting the relationship between PVAT dysfunction and CVD with a focus on potential mechanisms by which PVAT contributes to obesity-related CVDs are pointed out.

## Introduction

Obesity is a fast-growing problem that is reaching epidemic magnitudes worldwide, affecting both children and adults (Ogden et al., 2016; Lim et al., 2017). This condition is defined as a disproportionate body weight for height with excessive fat accumulation that is frequently accompanied by mild, chronic, systemic inflammation (Gonzalez-Muniesa et al., 2017). There is emerging body of scientific, medical, and behavioral data showing that central deposition of adipose tissue is associated with elevated risk of morbidity and mortality due to several cardiovascular complications, including stroke, congestive heart failure, myocardial infarction, and cardiovascular death, and this is independent of the association between obesity and the components of the metabolic syndrome and other cardiovascular risk factors (Arnlöv et al., 2010; Williams et al., 2015). Previous support for this understanding was provided by the American Heart Association in 1998, which has reclassified obesity as a major, modifiable risk factor for coronary heart disease (CHD) (Eckel and Krauss, 1998).

Besides being considered the largest energetic reservoir in the body, white adipose tissue (WAT) has been recognized as a remarkably complex endocrine organ that produces and secretes several substances with endocrine, paracrine, and autocrine functions, acting as a major regulator of systemic energy homeostasis (Rosen and Spiegelman, 2014). In obesity, adipose tissue may become dysfunctional and fail to appropriately expand to store the excess energy. This results in ectopic fat deposition in other tissues that regulate metabolic homeostasis (Tchoukalova et al., 2010). WAT expansion has been associated with numerous local consequences, including inflammation (Apovian et al., 2008), fibrosis (Henegar et al., 2008), hypoxia (Jiang et al., 2011), dysregulated adipokine secretion (Jernås et al., 2006; Skurk et al., 2007), and disrupted mitochondrial function (Heinonen et al., 2015). The whole-body consequences of WAT dysfunction include abnormal glucose and lipid metabolism, insulin resistance, increase in blood pressure, coagulation, fibrinolysis, inflammation, and endothelial dysfunction, all of which provide important mechanisms linking obesity to cardiovascular disease (CVD).

In addition to abdominal adiposity, recent evidence indicates that perivascular adipose tissue (PVAT) produces and releases a wide variety of adipokines and other factors that exert a paracrine influence on the vascular function, not only in veins and conductance arteries, but also in vessels of smaller caliber, which are essential in the regulation of blood pressure (Iozzo, 2011; Aghamohammadzadeh et al., 2012; Malinowski et al., 2013; Szasz et al., 2013). Although PVAT has been considered an inherent component that provides structural support to the vessels, now it is clear that this tissue possesses the dynamic capacity to mobilize near vessels with the potential for cellular communication and control of vascular function (Sacks and Fain, 2007; Chatterjee et al., 2009). Local accumulation of perivascular adipocytes has been consistently associated with the development of cardiometabolic complications in obesity (Chang et al., 2013; Lim and Meigs, 2014). In fact, adipose tissue surrounding the heart has been clinically associated with coronary artery disease (Cheng et al., 2008; Clément et al., 2009; Company et al., 2010), which reinforces the evidence that local adipose tissue accumulation can constitute an important regulator of cardiovascular function and a mediator of the development and progression of CVDs. The present review provides a comprehensive overview on the role of PVAT within the scope of adipose tissue as a major contributing factor to obesity-associated cardiovascular risk. We will also highlight the relevant clinical studies documenting the relationship between PVAT dysfunction and CVD and the potential mechanisms by which PVAT contributes to obesity-related cardiovascular complications.

## Obesity and cardiovascular risk

Over the past decades, an explosive increase in overweight and obesity prevalence has taken place in most of the high-income countries (Vandevijvere et al., 2015). Several recent reviews have also shown significant increases in the prevalence of these conditions in low- and middle-income countries, affecting both men and women, adults and children (Gupta et al., 2012; Popkin and Slining, 2013; Sayon-Orea et al., 2013; Yatsuya et al., 2014). Although there are great variations in the prevalence and trends of overweight and obesity among different regions, the numbers have been projected to further increase in coming years (Gupta et al., 2012; Popkin and Slining, 2013; Sayon-Orea et al., 2013; Yatsuya et al., 2014; Poobalan and Aucott, 2016). The percentage of adults with a body mass index (BMI) ≥25 kg/m^2^ between 1980 and 2013 increased in men and women from 28.8% (95% UI: 28.4–29.3) to 36.9% (36.3–37.4), and from 29.8 to 38%, respectively. Among children and adolescents, in 2013, 23.8% (22.9–24.7) of boys and 22.6% (21.7–23.6) of girls were either overweight or obese in developed countries (Ng et al., 2014). The prevalence rate in developing countries elevated from 8.1% (7.7–8.6) to 12.9% (12.3–13.5) for boys and from 8.4% (8.1–8.8) to 13.4% (13.0–13.9) in girls, in 2013. The World Health Organization (WHO) estimated that more than 2.1 billion adults were overweight or obese globally in 2014. By 2030, estimations predict that 57.8% of the adult population will have a BMI of 25 kg/m^2^ or higher (Kelly et al., 2008; Finkelstein et al., 2012). As such, the adverse health consequences of obesity, particularly the burden of CVDs are expected to increase in coming years.

In adults, obesity generally presents as an excess of body weight and adipose tissue, which is clinically assessed by the BMI, calculated by the weight in kilograms divided by the height in square meters (Garrow and Webster, 1985). The idea that BMI is associated with higher all-cause morbidity/mortality risk is supported by a wealth of epidemiological and clinical data (Borrell and Samuel, 2014; Yang et al., 2016; Kong et al., 2017). However, a recent comprehensive estimation, resulting from a systematic review on the association of all-cause mortality in adults with BMI categories used in the United States and internationally, demonstrated that while grades 2 and 3 obesity are both associated with significantly higher all-cause mortality, overweight (defined as a BMI of 25 – <30) is associated with significantly lower overall mortality relative to the normal weight category. Furthermore, the authors did not find significant excess mortality associated with grade 1 obesity (BMI of 30 – <35) (Flegal et al., 2013), suggesting that cardiovascular risk and mortality are not simply associated with the amount of adipose tissue.

Adipose tissue is dispersed throughout the body in discrete depots ranging from 5 to 60% of total body weight (Cinti, 2001). More than 80% of the tissue is found subcutaneously, mainly in the abdominal, gluteal and femoral areas. Visceral adipose tissue and smaller depots close to organs represent the remaining 10–20% of total body fat in men and 5–10% in women (Lee et al., 2013). It has become increasingly evident that the regional body fat distribution, the size of each depot, the depot-related differences in adipose tissue function, and the balance between them are important for the individual cardiometabolic risk. For example, a peripheral adiposity in upper and lower extremities is favorable while the truncal adipose tissue deposition, which includes subcutaneous fat in thoracic and abdominal region and the fat in intrathoracic and intraabdominal regions, is detrimental and associated with increased mortality (Garg, 2004; McLaughlin et al., 2011). Moreover, for any given BMI-value, there is significant variability with some lean individuals developing disease and others exhibiting a better metabolic profile than expected for their adiposity profile (Samocha-Bonet et al., 2012). Lapidus et al. in 1980s, reported a stronger association of waist-to-hip circumference ratio, which reflects abdominal fat, with a 12-year incidence of myocardial infarction, angina pectoris, stroke, and death, when compared to other anthropometric measures (Lapidus et al., 1984). Subsequently, many clinical studies have established the superior capability of waist-to-hip ratio measure to predict CVD risk when compared to BMI.

The large-scale randomized factorial clinical trial study ADVANCE (Action in Diabetes and Vascular disease: Preterax and Diamicron-MR Controlled Evaluation) examined the relative importance of different adiposity markers in predicting CVD risk in a population of individuals with type 2 diabetes (Czernichow et al., 2011). After 9.1 years of follow up, investigators reported that waist-to-hip ratio is the best predictor of cardiovascular events and mortality in patients with type-2-diabetes while the BMI is the worst. The INTERHEART study, a large multiethnic case-control study of acute myocardial infarction, also evidenced a greater contribution for waist-to-hip ratios accounting for most of the risk of myocardial infarction when compared to BMI, in both sexes and at all ages (Yusuf et al., 2004). A meta-analysis using data pooled from 15 prospective studies that included more than 258,000 subjects reported a progressive increase in cardiovascular risk accompanying the increase in waist circumference and waist-to-hip ratios (de Koning et al., 2007). Specifically, every 1 cm of increase in waist circumference was associated with a 2% increased relative risk of cardiovascular event and a 0.01 increase in waist-to-hip ratio was associated with a 5% increase in risk of future CVD for both men and women. The Nurses' Health Study, one of the largest and longest studies to date that measured abdominal obesity, confirmed these findings using the waist circumference measure (Zhang et al., 2008). The investigators reported that during 16 years of follow-up in US women, elevated waist circumference was associated with significantly increased CVD mortality even among normal-weight women.

As obesity is strongly linked to comorbid conditions, including type 2 diabetes, hypertension, hypercholesterolemia, hypertriglyceridemia, and non-alcoholic fatty liver disease, these risk factors have been considered important intermediate steps in the causal relationship between obesity and CVD risk. From this perspective, there has been considerable debate about whether the adjustment for these risk factors in statistical models is necessary for the true absolute risk predicting CVD risk or whether such adjustments, rather than controlling for, may increase the overall risk of bias. In fact, the increased cardiovascular risk attributed to weight gain persists even after adjustment for the frequently observed co-existing risk factors. In 1983, a 26-year follow-up of participants in the Framingham Heart Study reported that this risk was particularly apparent for heart failure, but it was also identified with CHD, stroke, and death from CVD. Kenchaiah and colleagues also provided unequivocal evidence in 2002, using updated Framingham data. According to authors, approximately 11% of heart failure cases among men and 14% among women are attributable to obesity alone, suggesting that efforts to promote optimal body weight may reduce the risk of heart failure (Kenchaiah et al., 2002). A careful review of these studies further supports the idea that composition of adipose tissue is important for cardiovascular risk stratification and characterizes visceral adipose tissue as an incomparable pathogenic depot that confers risk beyond its contribution to overall adiposity. These constraints that govern the outcomes of obesity are described in a recent review by Mahmood et al. (2014) and emphasizes the importance of adipose tissue depots as potential components in assessing and predicting cardiometabolic risk.

## Systemic adipose tissue dysfunction and vascular function in obesity

The WAT is no longer considered to be only a passive tissue for the storage of excess energy in the form of fat. There is now compelling evidence that this tissue acts as an endocrine organ that produces and releases biologically active compounds that regulate metabolic and cardiovascular homeostasis and undergoes pathological expansion during obesity (Waki and Tontonoz, 2007; Coelho et al., 2013). In addition to the better-known WAT, mammals also exhibit brown adipose tissue (BAT). Functional BAT in adult humans is localized to areas close to the clavicular, periaortic, cervical, and suprarenal regions (Cypess et al., 2009). BAT is an energy-expending organ that produces heat, and it is essential for adaptive thermogenesis. Adipocytes from BAT possess large numbers of mitochondria that contain a unique protein called uncoupling protein 1 (UCP1), which is a proton channel within the inner mitochondrial membrane involved in the dissipation of the proton motive force that is normally used to drive the synthesis of cellular ATP. Consequently, the energy in the mitochondrial electrochemical gradient is released in the form of heat (Cannon and Nedergaard, 2004). Indeed, evidence from clinical and experimental studies indicate that BAT activation increases thermogenesis (Lidell and Enerback, 2010). Recent reports have also unequivocally demonstrated that BAT exerts significant impact on whole-body energy homeostasis and that its activity profoundly influences body weight (Fruhbeck et al., 2009). In fact, overweight and obesity have been associated with lower BAT activity (Vijgen et al., 2011), which is increased after weight loss (Vijgen et al., 2012). Together with data obtained from rodent models (Vegiopoulos et al., 2010; Seale et al., 2011; Kiefer et al., 2012), these reports have drawn attention to BAT as an attractive target for the treatment of obesity and associated metabolic diseases. Hence, potent or permanent interventions targeting on induction of brown phenotype in adipose tissue depots will facilitate long term studies to investigate their precise role in driving metabolic diseases and to determine their potential to attenuate cardiovascular risk. However, it is discernible that such a critical approach will require layers of specificity, beyond the targeting of individual components of WAT, considering the possibility that factors involved in browning of specific adipose tissue depots may not share similar regulatory network.

**Figure 1:**
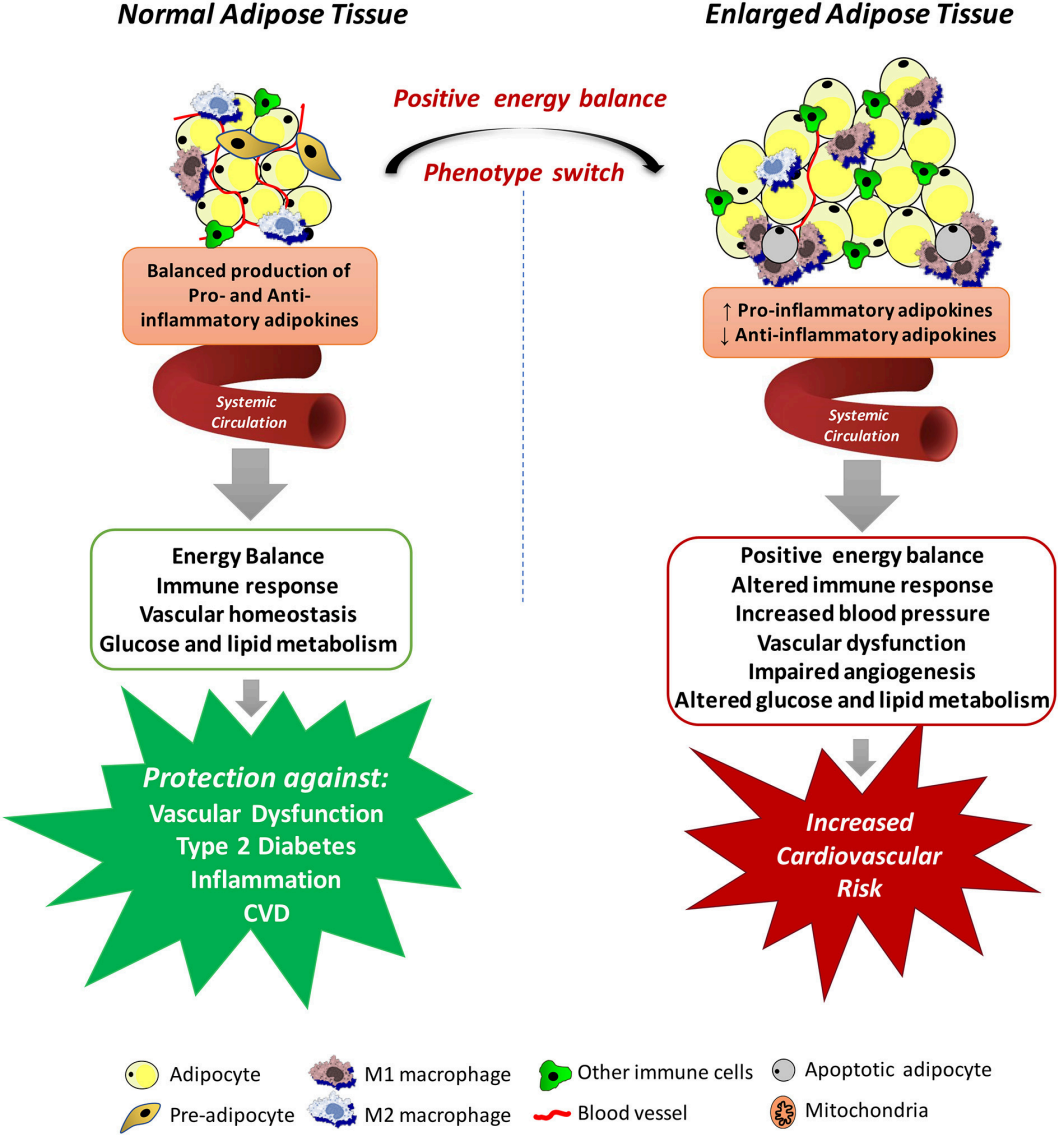
Mechanisms by which systemic adipose tissue dysfunction could lead to the development of vascular dysfunction in obesity. Adipose tissue produces various chemokines, cytokines and hormones, which are secreted into the circulatory system and act in several physiological processes, including energy balance, immune responses, blood pressure, vascular homeostasis and angiogenesis, glucose and lipid metabolism. Expansion of the adipose tissue leads to necrotic and/or apoptotic cell death and this is paralleled by infiltration of activated macrophages, increased production of pro-inflammatory adipokines and reactive oxygen species, promoting a state of systemic endothelial cell activation and vascular dysfunction, all of which may contribute to the elevated cardiovascular risk associated with obesity.

A highly relevant factor for the understanding of the cardiovascular impact of increased adipose tissue mass is the recognition that adipocytes are endocrine and paracrine determinants of vascular function. The great question that arises from these observations is how excessive accumulation of adipose tissue could lead to the development of such dysfunction even at a distance. The answer to this question is provided by the evidence that adipose tissue is involved in the production of various proteins, collectively called adipokines (including chemokines, cytokines, and hormones) (Kershaw and Flier, 2004), which are secreted into the circulatory system and act in several physiological processes, including energy balance, immune responses, blood pressure, vascular homeostasis and angiogenesis, glucose and lipid metabolism, and even insulin sensitivity (Prins, 2002), all of which may contribute to the elevated cardiovascular risk associated with obesity (Weyer et al., 2000; Wang et al., 2005). The positive energy balance induces an expansion and remodeling of the adipose tissue that is initially driven by adipocyte hyperplasia mediated by the recruitment and proliferation of adipogenic progenitors followed by an increase in adipocyte hypertrophy (Hausman et al., 2001; Spalding et al., 2008), which lead to dysregulated secretion of adipokines and increased release of free fatty acids (Figure 1).

Although the altered secretory function of adipose tissue is consistent with the idea that chronic obesity-related low-grade inflammation in adipose tissue is involved in the metabolic complications of obesity, it is important to consider that adipocytes in adipose tissue are not the only culprit. Regardless of the mechanisms of adipose tissue expansion, positive energy balance eventually leads to necrotic and/or apoptotic cell death and this is paralleled by quantitative and qualitative changes in the cellular composition and the phenotype of individual cells within adipose tissue (Weisberg et al., 2003). For example, adipose tissue from obese individuals is infiltrated by numerous activated macrophages, leading to increases in both absolute macrophage number and the relative level of macrophage-to-adipocyte ratio. In addition to this quantitative change, the macrophage phenotype is also altered by the obesity state. Macrophages that accumulate in adipose tissue of obese organisms tend to express genes associated with increased production of pro-inflammatory cytokines, express inducible nitric oxide synthase (iNOS), and produce high levels of reactive oxygen species (ROS), and nitrogen intermediates (Lumeng et al., 2007). Thus, it has become evident that interactions between the different cell types from enlarged and inflamed adipose tissue contribute to its overall impact on obesity-related disorders. In fact, some evidence exists that the release of some adipokines is even higher from the cells of the stroma-vascular fraction than from adipocytes (Chavey et al., 2009; Hamaguchi et al., 2012).

Adipose tissue dysfunction may also promote a state of systemic endothelial cell activation through the endocrine actions of inflammatory adipokines (Skurk et al., 2007; Dulloo et al., 2010). The impact of these adipokines on vascular function is not limited to the momentary regulation of the release of endothelium-derived vasoactive factors or regulation of vascular smooth muscle tone. Some adipokines may also profoundly affect local growth, migration, and inflammatory processes (Miao and Li, 2012). Obviously, these variety of products secreted by the adipose tissue, without even reporting the entirety of released adipokines, have provided promising possibilities for identifying novel biomarkers associated with obesity and the cardiovascular complications associated with this condition (Bagi et al., 2012; Barton et al., 2012). Although these studies set several adipokines into their functional context enhancing our current knowledge of the endocrine function of the adipose tissue, the identified proteins must be further validated regarding their expression, secretion and function. Translation of protein profiling results into clinical use will contribute to validate novel adipokines potentially representing a link between obesity and human disease. In this regard, studies must move from characterizing protein abundances to elaboration of their functional effects within cellular networks. However, since human genetic variability is estimated to contribute variations in the capacity of different adipose tissue depots to store and release fatty acids and to produce adipokines (White and Tchoukalova, 2014), and many of the direct genetic achievements are still being investigated for their causal impact on phenotypic outcomes, it is prudent to interpret both positive and negative results with caution. Integrated approaches including the analysis of multiple scientific fields will certainly enhance the understanding of this new physiological concept of interorgan crosstalk in the context of obesity.

## Perivascular adipose tissue effects on vascular function

Besides the endocrine role of adipose tissue, mediated by adipokines, a major emerging concept indicates that ectopic fat depots surrounding the heart and almost all systemic blood vessels, can also directly affect the cardiovascular function (Chang et al., 2013). These include: the PVAT, that is, the fat immediately adjacent to the adventitia of almost all arteries; the epicardial adipose tissue, which refers to the fat depot laying on the surface of the myocardium surrounding the coronary arteries; and the pericardial adipose tissue, the fat depot located between the visceral and parietal pericardium (Iozzo, 2011). Notably, this anatomical proximity of PVAT has an enormous influence on the cardiovascular system and highly vascularized organs (e.g., the kidney). In fact, over the past years, clinical and experimental evidence accumulated recognizing that PVAT not only stores triacylglycerols/triglycerides and free fatty acids, participating in energy metabolism, but also secretes a wide variety of biologically active molecules, including adipokines, such as leptin, adiponectin, chemerin, visfatin, resistin, tumor necrosis factor alpha (TNF-α), interleukin-6 (IL-6), interleukin-18 (IL-8), monocyte chemoattractant protein-1 (MCP-1), and plasminogen activator inhibitor 1 (PAI-1), which modulate vascular tone (Maenhaut and Van de Voorde, 2011), smooth muscle cells migration and proliferation (Miao and Li, 2012), neointimal hyperplasia and formation (Takaoka et al., 2010; Schroeter et al., 2013), inflammatory responses and oxidative stress (Salgado-Somoza et al., 2010). Most of these properties converge on endocrine-related effects, but also on a direct, paracrine influence in CVD, not only on large arteries and veins, but also in small and resistance vessels, and skeletal muscle microvessels, considered to be of greatest importance in blood pressure regulation, as has been reviewed elsewhere (Szasz et al., 2013; Xia and Li, 2017).

The structural and physiological characteristics of PVAT vary according to its location. In the mesenteric arteries, PVAT resembles WAT, with less differentiated adipocytes, poor vascularization, a specific profile of cytokines production/secretion, and contains infiltrates of macrophages, fibroblasts and cells of the immune system (Guzik et al., 2013). On the other hand, recent studies have demonstrated that PVAT of the thoracic aorta exhibits features that resemble BAT rather than WAT (Fitzgibbons et al., 2011; Chang et al., 2012), including expression of genes highly or solely expressed in the BAT, the presence of multilocular adipocytes and high abundance of mitochondria. Like BAT, PVAT is activated by cold and generates heat. Interestingly, the cold-induced activation of thermogenesis in PVAT is accompanied by attenuation of the atherosclerotic process in apolipoprotein E deficient mice (ApoE^−/−^), whereas such protection is lost in mice where PVAT is absent (Chang et al., 2012), indicating that the thermogenic properties of PVAT also mediate its vascular protective effects.

Consistent with the findings that thoracic aorta PVAT shares common characteristics with BAT, our group has recently demonstrated a potential role of mitochondria in periaortic adipose tissue as a source of mediators that could be involved in the modulation of vascular contraction along with the PVAT-derived relaxing factors (Costa et al., 2016). These are all exciting developments that have the potential to provide insight into new aspects of the specific nature and function of different adipose tissue depots while also places PVAT as a promising component of investigation regarding brown fat and its potential beneficial effects, including those on the vasculature. Further work is required to resolve interesting questions raised by these findings: are the precursor components of PVAT adipocytes distinct from those of white or BAT? Although the morphology and mRNA/protein profile of PVAT is evocative of classic BAT, is this tissue a true BAT? Recent studies have illustrated a remarkable possibility that the origin of PVAT adipocytes may so far be distinct from either white or brown adipocytes. Using a vascular smooth muscle cell (VSMC)-specific nuclear receptor activated by peroxisome proliferator-γ (PPARγ) deletion, Chang group generated mice completely devoid of PVAT in the aortic and mesenteric regions while both interscapular BAT and gonadal/inguinal/subcutaneous WAT remained intact, indicating that BAT, WAT, and PVAT have different origins (Chang et al., 2012). In this regard, the previous observation of Chatterjee's group demonstrated that *in vitro* differentiated human coronary artery perivascular adipocytes exhibit a distinct state of adipogenic differentiation and adipokine secretion when compared with adipocytes from subcutaneous or visceral adipose depots (Chatterjee et al., 2009), suggesting that PVAT exhibit a unique gene expression profile that underlies its role in vascular function. Although these well-established strategies provide extremely promising prospects, an unresolved question is the relative contribution of different brown and brown-like fat depots to metabolic and CVD in humans. Whether PVAT thermogenesis can be targeted for therapeutic purposes needs to be determined. It also seems clear that understanding the differences between PVAT depots, specifically, the functional analysis of bioenergetics in this tissue and its impact on systemic metabolism is a very promising approach in the context of metabolism and the etiology of the spectrum of cardiometabolic disorders.

The concept that PVAT influences vascular function, particularly contractile responses, dates from at least as early as the 1990s, when Soltis and Cassis described that PVAT retained and impaired the diffusion of pharmacological agents, justifying a reduction in the contractile response observed in vessels with this tissue (Soltis and Cassis, 1991). However, subsequent studies using several agonists of low retention in adipocytes also demonstrated such a decrease (Gollasch and Dubrovska, 2004). Löhn and his colleagues found that the vascular responses to angiotensin II (Ang II), serotonin (5-HT), and phenylephrine (PE) are reduced in intact aortas with PVAT, suggesting that periaortic adipose tissue regulates vascular tone by the release of transferable adventitia-derived relaxing factors (ADRF), now defined as PVAT-derived relaxant factors (PVRF) (Lohn et al., 2002). The anti-contractile effect of PVRFs under physiological conditions has been described in several species and is mediated by different mechanisms depending on the vascular bed. Convincing evidence has demonstrated that VSMC potassium (K^+^) channels play a critical role in mediating the relaxation responses to PVRFs. The study of Löhn showed that the anti-contractile effect of periadventitial fat was reduced by inhibition of ATP-dependent K^+^ channels and by the tyrosine kinase inhibitor genistein (Lohn et al., 2002). Following these findings, subsequent studies demonstrated that PVAT-derived transferable factors produce vasorelaxation by opening of voltage-dependent K^+^ (Kv) channels (Dubrovska et al., 2004; Verlohren et al., 2004; Gao et al., 2005; Fesus et al., 2007). Schleifenbaum's group further explored the involvement of the Kv-subfamily on PVRFs actions. The authors demonstrated that the voltage-activated family of K^+^ channels KCNQ (Kv7), which are not targeted by endothelium-derived relaxing factors (EDRF), play significant role in the vasorelaxation induced by PVRFs, which is at least in part mediated or modulated by hydrogen sulfide (H_2_S). Although the KCNQ channel subtype involved in the PVRF effects has not been identified so far and clear evidence for direct activation of KCNQ channels by PVRF is still missing, the opening of these channels by pharmacological agents restores the diminished anti-contractile effects of perivascular fat in spontaneously hypertensive rats, thus suggesting its pivotal role in perivascular regulation of vascular tone (Schleifenbaum et al., 2010).

Although we are unable to cover the topic in detail in this manuscript, it is important to note that many other mechanisms involved in PVRF-induced anti-contractile effects have been described. Lu and collaborators, by using inferior vena cava rings in the absence and presence of PVAT and endothelium, found that endothelium removal abolishes PVAT anti-contractile response. The same group of researchers has suggested that PVAT releases Ang 1–7, which, by acting on receptors in the endothelium, leads to nitric oxide (NO) release and activation of Kv channels with subsequent vascular relaxation (Lu et al., 2011). PVRF may also act through endothelium-independent mechanisms involving hydrogen peroxide (H_2_O_2_) production and subsequent activation of guanylyl cyclase (sGC) (Gao et al., 2007).

In addition to the vasodilator effects, there is also considerable evidence of contractile function mediated by PVAT. Soltis and Cassis reported the critical role of adipocyte-derived Ang II in PVAT-mediated potentiation of electrical stimulation-induced contraction in rat mesenteric arteries (Soltis and Cassis, 1991). This effect was subsequently shown *in vivo* systemically in rat mesenteric PVAT (Lu et al., 2010). Gao et al. also reported that PVAT enhances the contractile response of mesenteric arteries to perivascular nerve stimulation through superoxide anion (${\text{O}}_{2}^{-}$) production (Gao et al., 2006). The study of Payne documented that PVAT impairs coronary endothelial function in response to bradykinin both *in vitro* and *in vivo*, implicating local adipose tissue in the initiation and pathogenesis of coronary vascular disease (Payne et al., 2008). In particular, the authors documented that adipose tissue-derived factors diminish endothelial NO production through the direct inhibition of the enzyme NOS. Following these findings, the authors demonstrated that periadventitial adipose tissue-derived factors impair coronary endothelial NO production via a PKC-β-dependent, site-specific phosphorylation of endothelial NOS (eNOS) at Thr^495^ (Payne et al., 2009). Some adipose tissue-derived factors, such as leptin (Knudson et al., 2005), resistin (Dick et al., 2006), and TNF-α (Picchi et al., 2006), secreted under conditions of inflammation, can also attenuate vasodilatation, and these factors are produced by PVAT.

In the last years, several studies identified additional components involved in the contractile effects of PVAT, which have been reported especially in the context of obesity and CVD. One example of this is the study of Meyer's group, which demonstrated that PVAT controls arterial smooth muscle tone by releasing an “adipocyte-derived contracting factor” (ADCF) formed by cyclooxygenase (COX), which mediates the contractile effects of PVAT in obesity (Meyer et al., 2013). Another powerful example is the work of Watts et al. illuminating a potential role for the adipokine chemerin as an endogenous mediator that is responsible for vasoconstriction in obesity (Watts et al., 2013). In line with these observations, our group demonstrated that incubation of isolated vessels with chemerin for 24 h increases arterial sensitivity to endothelin-1 (Lobato et al., 2012). Thus, it is clear that PVAT is a complex, active organ with several functions beyond mechanical protection for the underlying vasculature and the current literature provides substantial evidence that this tissue produces many putative vasoactive factors that may influence vascular function and obesity-related vascular injury. However, most of these characterization studies have been carried out on vessel rings isolated from animal models, in the presence or absence of the PVAT layer. This poses an important and unresolved question regarding how much of these results can be translated *in vivo*, especially concerning human physiology. A thoughtful consideration of the implications of manipulating PVAT function to treat chronic vascular diseases is therefore warranted.

## Perivascular adipose tissue dysfunction as a major contributing factor to obesity-associated vascular dysfunction

**Figure 2:**
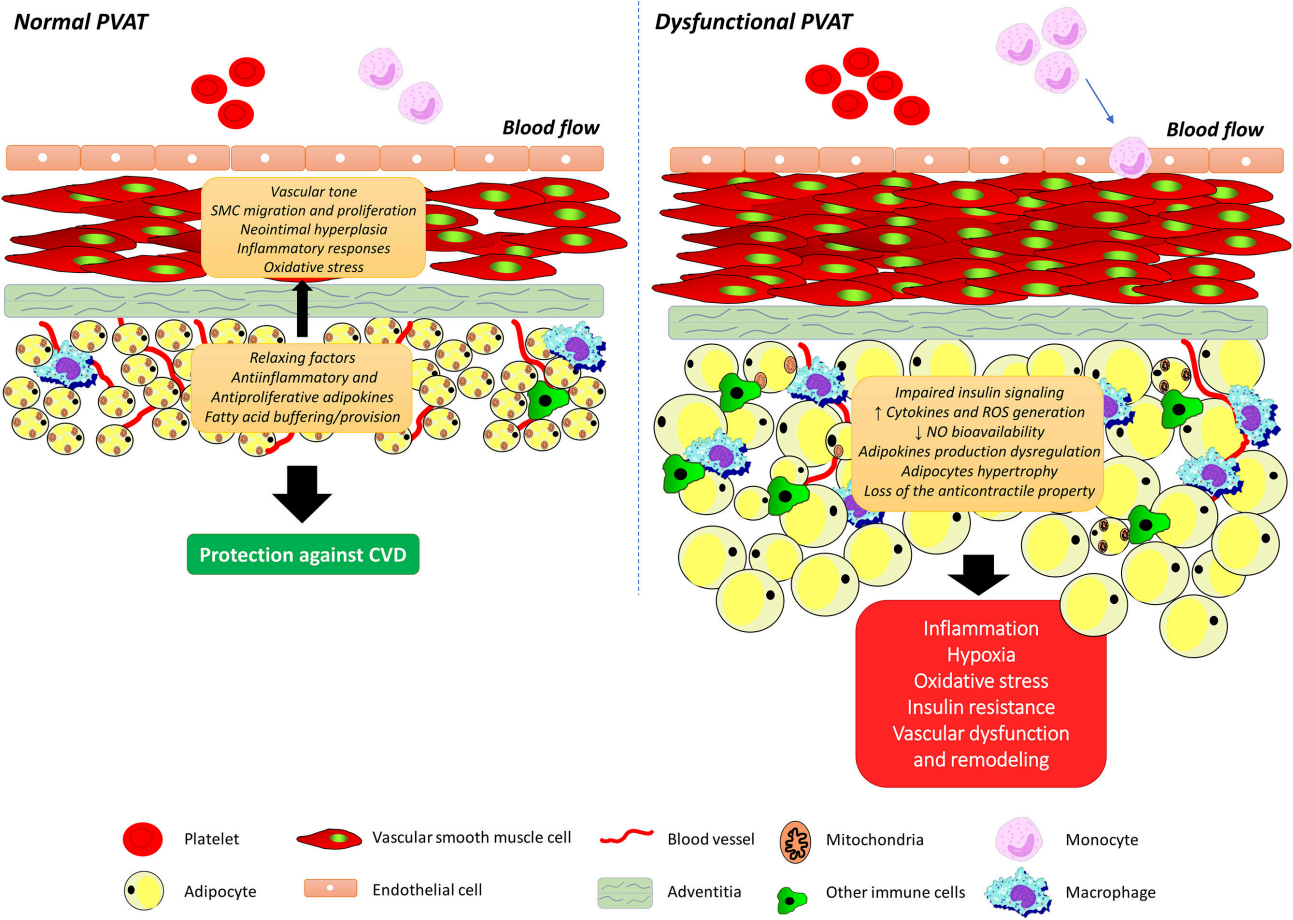
Perivascular adipose tissue dysfunction and obesity-associated vascular dysfunction. Perivascular adipose tissue (PVAT) secretes a wide variety of biologically active molecules, including the adipokines, which modulate vascular tone, smooth muscle cells migration and proliferation, neointimal hyperplasia, inflammatory responses, and oxidative stress. Obesity is associated with structural and functional changes in PVAT, leading to an imbalance in favor of vasoconstrictor and pro-inflammatory substances, adipocytes hypertrophy as well as changes in insulin signaling pathways. SMC, smooth muscle cell; ROS, reactive oxygen species; NO, nitric oxide.

As the understanding of the molecular mechanisms that underlie the connections between PVAT and vascular function progress, the ultimate goal is to establish the integrated role of this vascular component for obesity-related vascular dysfunction. Like observed with the total adipose tissue, PVAT mass is increased throughout the vasculature in both animal models and humans with obesity (Greenstein et al., 2009; Marchesi et al., 2009; Ketonen et al., 2010; Lehman et al., 2010). Considering that the anti-contractile influence of PVAT is directly dependent on its volume (Verlohren et al., 2004; Gao et al., 2006), it would be discernible that such an increase would be associated with enhancement of PVAT anti-contractile effects. However, available support for our understanding of the connection between PVAT and vascular dysfunction in obesity comes from the findings that obesity is associated with structural and functional changes in PVAT, leading to an imbalance in favor of vasoconstrictor and pro-inflammatory substances, as well as changes in the signaling pathways in the vessel and that this condition interferes with vascular function (Figure 2). Numerous studies have since validated this postulate (reviewed in Xia and Li, 2017). In line with this observation, Gao et al. (2005) demonstrated that increased adiposity causes an alteration in the modulatory function of PVAT on vascular relaxation response. New Zealand Obese (NZO) mice, which present most symptoms of the metabolic syndrome and a greater amount of PVAT, also show a reduction in the anti-contractile effect of PVAT (Fesus et al., 2007). Higher periaortic fat mass in rats treated for 6 months with high fat diet (HFD) lead to reduced endothelium-dependent relaxation due to downregulation of AMP-activated protein kinase (AMPK) and eNOS in the aorta with a concurrent upregulation of the mTOR ripamycin target, which negatively regulates the AMPK-eNOS pathway (Ma et al., 2010). Another important milestone toward our basic understanding of the relationship between PVAT dysfunction and obesity-associated vascular dysfunction was the demonstration of changes in the proteomic profile of 186 proteins in PVAT of coronary arteries, which correlate with increased contractile effect and the activation of calcium (Ca^2+^)-dependent signaling pathways in VSMCs from Ossabaw pigs (Owen et al., 2013).

These considerations aside, the diverse aspects of PVAT influence on vascular dysfunction in obesity could be also framed by examining its high relationship with oxidative stress. ROS, such as ${\text{O}}_{2}^{-}$ and H_2_O_2_, play important roles in the modulation of vascular function by the PVAT. H_2_O_2_ is a vasoactive substance that induces both contractile and relaxing responses by different mechanisms depending on its concentration, the type and contraction state of the vessel, and the animal species. H_2_O_2_-induced relaxation may be endothelium dependent as a result of increased NO release secondary to K^+^ channel activation. In addition, H_2_O_2_ induces endothelium-independent relaxation through the direct opening of K^+^ channels in VSMC, by the oxidation of its cysteine residues, as well as by the direct activation of the sGC enzyme (Ardanaz and Pagano, 2006). The contractile effect mediated by H_2_O_2_ occurs due to the direct activation of the enzyme COX and an increase of intracellular Ca^2+^. Evidence indicates that ROS accumulation activates contraction pathways related to MAPKs, with increased ERK 1/2 phosphorylation (Peters et al., 2000). In addition, H_2_O_2_ activates the Rho kinase pathway, favoring vascular contraction (Ardanaz and Pagano, 2006). In fact, the Rho kinase pathway is important not only in the contraction of VSMC, but also in increased proliferation and cell migration, which has attracted much attention in relation to its role in the pathogenesis of CVDs (Loirand et al., 2006). Considering that ${\text{O}}_{2}^{-}$ induces vascular contraction while H_2_O_2_ has dual effects, the final result of the action of these ROS will depend on the relative balance between the production and the release of these factors by the PVAT. Ketonen et al. demonstrated in C57BL/6J mice fed a HFD that the reduction in endothelium-dependent relaxation occurs due to oxidative stress in PVAT, characterized by an increase in ${\text{O}}_{2}^{-}$ and H_2_O_2_ production (Ketonen et al., 2010).

The NAD(P)H oxidase, which is the major source of ${\text{O}}_{2}^{-}$ in the vasculature, is expressed in PVAT of rat mesenteric arteries and contributes to increased contractile response to perivascular nerve stimulation (Gao et al., 2006). Indeed, the expression of p67phox subunit of the NAD(P)H oxidase complex is increased in periaortic adipose tissue from short-term fed HFD obese mice (60% cal from fat) and this is accompanied by enhancement of both ${\text{O}}_{2}^{-}$ and H_2_O_2_ levels (Ketonen et al., 2010). Increased NAD(P)H oxidase activity and ${\text{O}}_{2}^{-}$ production associated with decreased expression of total SOD activity and extracellular superoxide dismutase (ecSOD) were described in the PVAT of these animals. The changes were accompanied by a reduction in eNOS expression and NO production in the PVAT (Gil-Ortega et al., 2014). Similarly, in NZO mice, increased ${\text{O}}_{2}^{-}$ formation and reduced SOD expression in PVAT contribute to vascular dysfunction by reducing the anti-contractile effect of this tissue (Marchesi et al., 2009). Gil-Ortega et al. reported that long-term HFD induces substantial reduction in ec-SOD expression and total SOD activity, an increase of NOX activity and ${\text{O}}_{2}^{-}$ release from the mesenteric PVAT, suggesting that the imbalance between antioxidant and pro-oxidant mechanisms in PVAT might contribute to vascular oxidative stress, thus aggravating endothelial dysfunction (Gil-Ortega et al., 2014). Marchesi et al. have also shown that the loss of PVAT anti-contractile properties in NZO mice might be associated with increase in both ${\text{O}}_{2}^{-}$ and NAD(P)H oxidase activity (Marchesi et al., 2009). Uncoupling of eNOS in PVAT, which contributes not only to increase ROS formation but also to decrease NO bioavailability, was also recently described as a novel mechanism underlying the vascular dysfunction in diet-induced obesity (Xia et al., 2016).

The mitochondrial electron transport chain (mETC), a significant source of ROS, has also been pointed as an integral component implicated in the physiological regulation of vascular function by PVAT. We recently showed that ${\text{O}}_{2}^{-}$ production from the mETC is increased in PVAT during norepinephrine (NE)-induced aortic muscle contraction. ${\text{O}}_{2}^{-}$ is subsequently dismutated to H_2_O_2_ by manganese SOD (Mn-SOD), which, in turn, modulates VSMC contraction (Costa et al., 2016). Following these findings, we next provided unequivocal evidence linking mitochondria to PVAT-associated oxidative stress and the subsequent loss of the PVAT anti-contractile effects observed in experimental obesity (da Costa et al., 2017). Under these conditions, the increase in PVAT-mediated ROS generation becomes an important sign of increased vascular contraction. As PVAT has a similar phenotype to BAT, including the expression of UCP-1, which is necessary for non-shivering thermogenesis, as discussed above, and considering that local energy metabolism induced by changes in temperature affects vascular function and atherogenesis (Brown et al., 2014), it can be proposed that increased energy production in PVAT under obesity condition also affects vessel biology favoring the development of CVD.

Structural and functional modifications of PVAT in obesity can also induce vascular remodeling. Periaortic adipocytes significantly increase the growth rate of aortic smooth muscle cells from aged and obese Wistar Kyoto rats supplemented with HFD. This effect is abolished in the presence of proteinase K, but is maintained in the presence of filtered proteins with a molecular mass less than 100 kDa, indicating that PVAT releases soluble proteins that stimulate growth of VSMCs, which can further aggravate aging- and obesity-associated vascular diseases (Barandier et al., 2005). Finally, the paracrine actions of factors produced and released by PVAT can also change vascular insulin sensitivity (Meijer et al., 2013). Although the prospective association between PVAT dysfunction and diabetes has not been demonstrated so far, this possibility has been previously postulated by Yudkin et al. and remarkably illustrated by several subsequent studies (Yudkin et al., 2005). Meijer's group showed that local depots of PVAT, which surround resistance arteries in the muscle microcirculation, controls vascular responses to insulin through adiponectin secretion and subsequent activation of AMPK signaling (Meijer et al., 2013). The expression of adiponectin is significantly lower in the epicardial adipose tissue isolated from patients with CAD (Iacobellis et al., 2005). In humans, PVAT accumulation around the brachial artery was negatively correlated with insulin sensitivity and the post-ischaemic increase in blood flow. The association was independent of the presence of other cardiovascular risk factors, including age, sex, liver fat, BMI, and visceral adipose tissue (Rittig et al., 2008). Additionally, PVAT from obese mice inhibits insulin-induced vasodilatation, which can be restored by inhibition of the inflammatory kinase Jun NH (2)-terminal kinase (JNK) (Meijer et al., 2013). JNK may directly induce insulin resistance by a mechanism that involves phosphorylation of the insulin receptor substrate (IRS) 1, blocking the transduction signal produced by the insulin receptor (Sabio et al., 2008). Studies in humans and animal models have demonstrated an important upstream role of this protein in integrating inflammatory and metabolic function that were recently reviewed elsewhere (Hotamisligil, 2017).

These findings, in addition to the observations that local accumulation of adipose tissue is consistently related to decreased flow-mediated vasodilation (Albu et al., 2005), fasting insulin levels and insulin resistance in humans (Rittig et al., 2008), support the involvement PVAT-derived factors as paracrine, rather than endocrine mediators of both microvascular dysfunction and insulin resistance in obesity. There is also tremendous redundancy in these pathways that support the view that the association between PVAT and cardiometabolic risk factors might not simply be the consequence of overall adiposity, but potentially constitutes an additional risk factor. Considering that hyperglycemia and hyperinsulinemia can also directly impair vascular function and consequently glucose disposal, it is also possible to suggest a vicious cycle of PVAT dysfunction that contributes to and is exacerbated by the impairment in insulin homeostasis.

## Inflammation in perivascular adipose tissue: the link between obesity and cardiovascular disease?

In the last decades obesity has been associated with a moderate degree of inflammation in adipose tissue, a condition that results from chronic activation of the innate immune system. This type of inflammation, called metaflammation or metabolic inflammation is free of pathogens and orchestrated by metabolic cells in response to excess nutrients and energy (Hotamisligil, 2017). Initial support for this understanding came from the following observations: macrophages-derived TNF-α induces insulin resistance in adipocytes (Pekala et al., 1983); adipose tissue from obese individuals displays increased expression of inflammatory mediators (Hotamisligil et al., 1993; Uysal et al., 1997); and inflammation promotes disturbances in glucose metabolism. It is relevant to emphasize here the first demonstrations that macrophage infiltration occurs into the adipose tissue in obesity (Weisberg et al., 2003; Xu et al., 2003). These findings were the basis for elucidating the involvement of adipose tissue macrophages as direct modulators of metabolism, and led to the observations that the increased free fatty acids exposure in obesity promotes the polarization of macrophages resident in the adipose tissue toward a pro-inflammatory (M1-polarized) phenotype which can activate inflammatory pathways and impair insulin signaling (Hevener et al., 2007; Lumeng et al., 2007; Nguyen et al., 2007). The molecular mechanisms underlying these events include epigenomic alterations that determine macrophage sensitivity to metabolically driven inflammatory (metaflammatory) signals (Fan et al., 2016), and additional macrophage-secreted products, including the potent and pleiotropic immune mediator TNF-α, which attenuates insulin signaling in adipocytes (Li et al., 2016). Many other immune cells including dendritic cells, mast cells, eosinophils, and lymphoid cells also contribute to metabolic tissue homeostasis and to the control of glucose metabolism. Regarding the initiation of metaflammation, an emerging concept is that adipocyte hypertrophy causes local hypoxia, which, in turn, causes infiltration of macrophages and other immune cells (CD4^+^ and CD8^+^ T cells, natural killer T cells, and mast cells) in visceral adipose tissue (Roemeling-van Rhijn et al., 2013). This increase in cells of the immune system along with the proinflammatory cytokines in adipocytes may negatively regulate PPARγ activity, which is essential for both adipogenesis and maintenance of the tissue gene expression (Guri et al., 2008).

Intermittent inflammatory processes have also been observed in PVAT. These include increased migration of immune cells, altered production of pro- and anti-inflammatory cytokines, adipokines, and lipid mediators, as well as signaling through a plethora of immune receptors and intracellular signaling molecules. Importantly, this entire cascade and mediators have now provided highly promising evidence that PVAT inflammation plays a key role at various stages of CVD. One powerful example of this is the recent work illuminating a role for perivascular inflammation as an alteration that precedes atherosclerotic plaque formation and even the development of endothelial dysfunction and oxidative stress in ApoE^−/−^ mice (Skiba et al., 2017). Specifically, the authors found increased leukocyte infiltration in the perivascular tissue when compared to the vessel wall. Furthermore, it was demonstrated that PVAT or adventitial inflammation and infiltration with macrophages and T cells precede not only significant atherosclerotic plaque development, but also the impairment of endothelium-dependent NO bioavailability. In support of this finding, it was previously shown that major risk factors for atherosclerosis, including hypertension, hyperlipidemia or ty 2 diabetes promote perivascular inflammation before and during development of atherosclerosis (Galkina et al., 2006; Galkina and Ley, 2009; Sagan et al., 2012; Guzik et al., 2013).

Molecular mechanisms linking PVAT inflammation in obesity to CVD indicate several key components. These may include chemotactic migration of PVAT immune cells into adventitia, with release of cytokines, which can alter vascular function (Mikolajczyk et al., 2016). In this regard, it was previously demonstrated that human PVAT exhibits a strong chemotactic activity on monocytes, granulocytes, and T lymphocytes that is mainly mediated by the secretion of IL-8 and MCP-1, which are known proatherogenic chemokines and their production is increased in obesity. These mediators are likely to contribute to the infiltration of leukocytes at the interface between PVAT and adventitia of atherosclerotic aortas (Henrichot et al., 2005). Visceral adipose tissue accumulation also promotes an increase in the secretion of angiopoietin-like protein 2 (Angptl2), a pro-inflammatory factor derived from adipocytes and considered a key mediator of chronic adipose tissue inflammation and obesity-related systemic insulin resistance (Tabata et al., 2009). In fact, Angptl2 secreted by PVAT accelerates neointimal formation after endovascular injury in mice by increasing inflammation-related gene expression and by accelerating extracellular matrix degradation (Tian et al., 2013). In line with these experimental findings, the authors provided relevant clinical data that strongly suggest that PVAT-secreted Angptl2 plays a significant role in accelerating vascular inflammation by cooperating with pro-inflammatory TNF-α or counteracting the anti-inflammatory activity of adiponectin, potentially leading to development of atherosclerosis in humans (Tian et al., 2013).

PVAT inflammation may also be associated with altered release of adipokines and other adipocyte-derived relaxing factors (Antonopoulos et al., 2015; Woodward et al., 2017). Initial support for this understanding came from the findings that perivascular adipocytes from human and mice without atherosclerotic disease exhibit a heightened proinflammatory state and reduced adipocytic differentiation under basal conditions as compared with adipocytes derived from subcutaneous and visceral adipose depots, and that high-fat feeding causes further reductions in adipocyte-associated gene expression while upregulates proinflammatory gene expression (Chatterjee et al., 2009). Specifically, secretion of the anti-inflammatory adipokine adiponectin is markedly reduced, whereas that of proinflammatory cytokines IL-6, IL-8, and MCP-1, is markedly increased in perivascular adipocytes. These changes have also direct effects on the PVAT vasoactive properties, as evidenced in animal and human small-artery studies, where hypoxia and inflammation were shown to attenuate the local vasoactive properties of PVAT by oxidative stress (Greenstein et al., 2009).

In addition to the previously-mentioned adipokines, chemerin, a secreted protein originally revealed as a chemoattractant molecule for immature dendritic cells and macrophages (Wittamer et al., 2003) is now considered a novel adipokine that regulates adipogenesis, adipocyte metabolism (Bozaoglu et al., 2007; Goralski et al., 2007) and inflammation (Parolini et al., 2007; Cash et al., 2010). Chemerin acts through CMKLR1 (chemokine-like receptor 1) or ChemR23 (chemerin receptor 23), which is expressed in macrophages, dendritic cells, adipocytes and vascular cells (Zabel et al., 2005, 2006; Parolini et al., 2007). Chemerin is considered a biomarker for adiposity since its plasma levels strongly associate with BMI and is linked to obesity and metabolic syndrome, a cluster of metabolic disorders that increase the risk for diabetes and CVD (Bozaoglu et al., 2009; Li et al., 2014). Furthermore, serum chemerin levels are significantly elevated in morbidly obese patients and reduced with the weight loss after bariatric surgery. The strong decrease of chemerin after surgery was associated with an improvement in insulin sensitivity and blood glucose, which further support the key role of this adipokine in mediating metabolic alterations in obesity (Ress et al., 2010; Sell et al., 2010). Animal studies reported the parallel findings that knockout mice for the primary receptor for chemerin, ChemR23, exhibit reduced food consumption, weight gain, and adiposity (Ernst et al., 2012). Consistent with this phenotype, ChemR23 knockout mice also displayed lower fasting blood glucose and serum insulin levels.

Recent observations have also shown that chemerin is highly expressed in PVAT. Spiroglou et al. first detected chemerin expression in epicardial and periaortic adipose tissue and demonstrated a positive correlation of this adipokine with both aortic and coronary atherosclerosis (Spiroglou et al., 2010). Most recently, Watts and her co-workers reported that chemerin produced by periaortic PVAT stimulates vascular contraction through the receptor typically attributed to function only in immune cells. Moreover, arteries from obese or hypertensive mice or obese humans with dysfunctional endothelium demonstrated amplified contractions in response to chemerin (Watts et al., 2013). Although there is no empirical evidence linking specific chemerin signaling pathways with the PVAT inflammation, a bulk of animal and human studies exploring the immune functions of chemerin make this a very plausible possibility. CMKLR1 is expressed in several immune cell types known to accumulate in obese adipose tissue (Wittamer et al., 2003; Parolini et al., 2007; Cash et al., 2010). Sell and collaborators demonstrated that chemerin activates the NF-κB pathway and impairs glucose uptake in skeletal muscle cells (Sell et al., 2010). In human endothelial cells, it was shown a significant upregulation of chemerin receptor by pro-inflammatory cytokines such as TNF-α, IL-1β, and IL-6 (Kaur et al., 2010). Accordingly, circulating levels of chemerin are elevated in diseases associated with chronic inflammation, including obesity and the metabolic syndrome (Wittamer et al., 2003; Bozaoglu et al., 2009; Li et al., 2014). Our recent studies also point to a role of chemerin in regulating inflammatory processes. Chemerin was shown to decrease NO-dependent cGMP signaling, thereby reducing vascular relaxation in rat aorta, an effect related to increased generation of ${\text{O}}_{2}^{-}$, an important mediator of inflammation (Neves et al., 2014). In line with these findings, we demonstrated that chemerin, through Nox activation and redox-sensitive MAPKs signaling, exerts proapoptotic, proinflammatory, and proliferative effects in human vascular cells (Neves et al., 2015).

Taken together, these findings not only provide multiple layers of potential mediators for the inflammatory component of PVAT in obesity but also clearly demonstrate that bidirectional interactions between that the systemic metabolic inflammation and local immune components are critical considerations in determining the physiological and pathological vascular outcomes associated with obesity. The proximity of PVAT as a rich source of proinflammatory cytokines and other mediators together with the associated alterations in this tissue support the concept that specific changes of local adipose tissue depots contribute to disease processes in the neighboring vessel wall.

## Clinical measure of perivascular adipose tissue in obesity-associated cardiovascular risk assessment: finding the point

Some of the initial thoughts and considerations of the reciprocal relationship between PVAT and risk factors for CVD have been provided by clinical findings showing an association between both perivascular and epicardial adipose tissue with the main anthropometric and clinical parameters of the metabolic syndrome (Iacobellis et al., 2003b). In fact, a very good correlation is observed between epicardial adipose tissue, assessed by echocardiography, and waist circumference, diastolic blood pressure, fasting plasma insulin, LDL cholesterol, and plasma adiponectin (Iacobellis et al., 2003b). Importantly, the association of insulin sensitivity and low adiponectin levels with the epicardial fat thickness is independent of BMI, suggesting that PVAT assessment might provide a more sensitive and more specific measure of the true visceral fat content (Iacobellis et al., 2003a). In support of these findings, a recent meta-analysis of published reports concluded that echocardiographic epicardial adipose thickness is significantly higher in patients with metabolic syndrome than in those without it (Pierdomenico et al., 2013).

Echocardiographic evaluation of epicardial adipose tissue thickness is considered a very reliable method to measure visceral adiposity, as first proposed and validated by Iacobellis et al. (2005). It is also relatively cheap and easy to perform as a screening test for assessment of patients suspected to be at risk for cardiovascular or metabolic outcomes. However, the technic provides only measurements of the regional thickness of epicardial adipose tissue and presents the risk to confuse pericardial fluid with adipose tissue since the fluid also shows up as relatively echo-free on an echocardiograph (Singh et al., 2007). The advances in imaging technology have enabled a more direct quantitative assessment of the local fat depots and have also been the basis for investigations into the possibility that PVAT acts as direct modulator of metabolism and cardiovascular function. From this perspective, clinical findings showed that volumetric quantification of epicardial and peri-coronary adipose tissue thickness by computed tomography, which provides a more accurate assessment due to its higher spatial resolution, is also positively related with parameters of obesity, such as BMI, waist circumference and the abdominal visceral adipose tissue mass (Gorter et al., 2008). In line with this, PVAT thickness, measured by computed tomography in the areas of right coronary artery, left anterior descending artery and left circumflex coronary artery is positively associated with waist circumference, waist-to-hip ratio, BMI, blood glucose, triglycerides, and systolic blood pressure. Epicardial adipose tissue is also linked to coronary calcification, a known marker of coronary atherosclerosis (de Vos et al., 2008).

In agreement with the above observations, abdominal periaortic adipose tissue and thoracic periaortic adipose tissue volume, measured by computed tomography, are consistently correlated with visceral abdominal fat, subcutaneous abdominal fat, waist circumference, and BMI in a random subset of participants from the Framingham Heart Study (Schlett et al., 2009). Thanassoulis et al. extended these findings by showing that both thoracic and abdominal periaortic adipose tissue volumes, measured using the same approach, are associated with higher aortic dimensions even after adjustment for other vascular and metabolic risk factors including global measures of obesity such as BMI (Thanassoulis et al., 2012). By contrast, the study of Rosito et al. found no significant associations between pericardial fat and CVD risk factors compared with visceral fat when both were considered in the same model. Nevertheless, the authors evidenced strong correlations between pericardial fat volume and metabolic risk factors even after adjusting for many potential confounders, including age, smoking, alcohol use, and physical activity. Furthermore, pericardial fat was found to be associated with coronary artery Ca^2+^ and abdominal aortic Ca^2+^ even after metabolic risk factors and visceral adipose tissue were accounted for, which is consistent with the postulation that pericardial fat may in fact exert a harmful perivascular effect on the coronary arteries (Rosito et al., 2008).

## Summary

There is tremendous wealth in the mechanisms that support the understanding of the relationship between excess adiposity and cardiovascular risk. The strongest evidence comes from the observations of basic science and translational studies regarding important physiological process that occur in adipose tissue and deleterious effects of relevant importance on vascular complications. It is interesting to consider the role that adipokines play among the mechanisms whereby adipocytes influence vascular function. However, there is considerably more work to be performed in both basic science and clinical areas to understand and reduce the enhanced CVD risk that is evident in the obese state. Clearly, these intriguing observations are not sufficient to answer the question whether increased PVAT mass represents a surrogate marker of cardiovascular risk or an independent pathogenic variable. However, outcome studies need to establish whether PVAT depots have prognostic significance and may therefore provide additional evidence for a causal relationship. Although the reduction of whole-body fat would be ideal among individuals who have obesity, a thoughtful consideration of the additional benefits of targeting fat depots at specific locations in individuals at greater cardiometabolic risk represents an alternative approach.

Further dissection of the vascular signaling pathways altered by PVAT-derived factors will likely reveal functional strategies for suppressing the negative effects of abnormal adipose tissue excess on CVD complications without altering the beneficial effects of normal fat depots. The challenges remaining in this field can be identified in two different areas. How does structural and functional changes of PVAT start in obesity and at what point does it become detrimental? What are the appropriate model systems and paths to elucidate the unknown mechanisms to prevent or treat human CVD? Regarding the contribution of new characterized adipokines, there are also several interesting and emerging concepts. Given these challenges, and the need for therapeutic approaches that do not permanently interfere with entire physiological effects of the PVAT, conveying the experimental insights into successful clinical interventions will require collaborative and diverse cross studies, new experimental approaches and integration of genetic variations with other environmental modifiers to establish links with complex cardiovascular phenotypes and facilitate successful translation to human disease.

## Author contributions

RC, KN, RT, and NL equally contributed to the conception of paper, drafting the manuscript, and approved its final version.

### Conflict of interest statement

The authors declare that the research was conducted in the absence of any commercial or financial relationships that could be construed as a potential conflict of interest.
